# Association of age-related decrease in intracellular-to-total water ratio with that in explosive strength of the plantar flexors: a cross-sectional study

**DOI:** 10.1186/s40101-022-00284-2

**Published:** 2022-03-26

**Authors:** Kosuke Hirata, Natsuki Iida, Akihiro Kanda, Mikio Shoji, Tsukasa Yoshida, Yosuke Yamada, Ryota Akagi

**Affiliations:** 1 Faculty of Sport Sciences, Waseda University, Tokorozawa-shi, Saitama, Japan; 2 College of Systems Engineering and Science, Shibaura Institute of Technology, Saitama-shi, Saitama, Japan; 3 Graduate School of Engineering and Science, Shibaura Institute of Technology, Saitama-shi, Saitama, Japan; 4 Mizuno Corporation, Suminoe-ku, Osaka, Japan; 5 Section of Healthy Longevity Research, National Institute of Health and Nutrition, National Institutes of Biomedical Innovation, Health and Nutrition, Shinjuku-ku, Tokyo, Japan

**Keywords:** Rate of torque development, Bioelectrical impedance spectroscopy, Muscle quality, Twitch contractile properties, Triceps surae, Time-to-peak torque

## Abstract

**Background:**

We aimed to investigate the association of age-related differences in the intracellular-to-total water ratio with explosive strength of the plantar flexors.

**Methods:**

A total of 60 young (21–33 years) and older (64–83 years) individuals were recruited. Intracellular- (ICW) and total-water (TW) content within the right leg was evaluated by bioelectrical impedance spectroscopy as indicators of muscle cell mass and whole muscle mass within the segment, respectively. ICW divided by TW (ICW/TW) was calculated as an index of the occupancy of muscle cells within whole muscle. Rate of torque development (RTD) and electromyography (EMG) activity during maximal voluntary isometric plantar flexion were measured as indicators of explosive muscle strength and neuromuscular activity, respectively. RTD was calculated from three time windows of 0–50, 50–100, and 100–200 ms. Time-to-peak torque (TPT) was assessed from evoked twitch contraction.

**Results:**

Compared with young participants, older participants showed lower ICW/TW (−7%, *P* < 0.001), RTD (−25 to −40%, *P* = 0.003 to 0.001), and longer TPT (+11%, *P* < 0.001). ICW/TW associated positively with RTD (*r* = 0.377 to 0.408, *P* = 0.004 to 0.001) and negatively with TPT (*r* = −0.392, *P* = 0.002), but not with EMG activity. RTD was associated positively with EMG for each time window (*r* = 0.527 to 0.607, *P* < 0.001).

**Conclusions:**

These results indicate that ICW/TW may be a useful predictor of the age-related decrease in RTD, and that the decrease in ICW/TW with age may reflect age-associated changes in intrinsic contractile properties.

## Background

Age-related decrease in explosive muscle strength (i.e., ability to achieve rapid muscle force development) is steeper than maximal muscle strength loss [1]. Since explosive muscle strength is related to the risk of falling [2], monitoring its decrease with age may be necessary. The rate of torque development (RTD) is defined as the slope of the time-torque curve and is a representative index of explosive muscle strength. RTD is determined by multiple factors, such as muscle mass, neuromuscular activity, and twitch contractile properties [3]. It is also known that fiber type composition is an influencing factor of RTD. Harridge et al. [4] suggested that higher type II fiber content leads to superior RTD. Since selective muscle atrophy occurs in type II fibers with age [5], an age-related shift in muscle fiber type composition is believed to be associated with a decrease in RTD.

Typical events of skeletal muscle aging not only involve a change in fiber type composition but also an increase in extramyocellular space, indicating a decrement of the occupancy of muscle cells within the skeletal muscle tissue [6]. Segmental bioelectrical impedance spectroscopy (S-BIS) can noninvasively and quickly assess intracellular- (ICW) and extracellular-water (ECW) content within skeletal muscles [7]. ICW strongly associates with maximal muscle strength irrespective of age [8], meaning that ICW can be equated to muscle cell mass [9]. Also, the ratio of ICW to total water (TW, the sum of ICW and ECW; ICW/TW) represents the proportion of muscle cells within whole muscle [10]. Indeed, previous studies have revealed that a reduction of ICW/TW occurs with aging [8], and that ICW/TW can explain the variability of muscle strength per unit size of older adults [11].

Considering that a decrease in type II fiber content [5] and an increase in extramyocellular space [8] are typical events of aging muscle atrophy, age-related differences in ICW/TW and RTD may be linked; however, to the best of our knowledge, this association has not yet been explored. Here, we aimed to cross-sectionally examine the association of age-related differences in ICW/TW with RTD in young and older individuals. Measurement parameters were S-BIS variables (ICW/TW and ICW), RTD, and factors affecting RTD, which included maximal muscle strength, neuromuscular activity, and twitch contractile properties [12]. We hypothesized that ICW/TW would positively relate to RTD. Plantar flexion RTD associated with balance ability in older adults [13], and an age-related decrease in plantar flexion RTD, is suggested to lead to a high risk of falling [2]. Hence, the right plantar flexors were investigated in the present study.

## Methods

### Participants

A priori power analysis was conducted to calculate the sample size for the correlation analysis using the G*Power statistical power analysis software (G*Power 3.1.7; Kiel University, Germany). We assumed a type 1 error of 0.05, a statistical power of 0.80, and an effect size of 0.35. This effect size was based on a previous study [14], which reported the Pearson correlation coefficients between RTD and the index of intramuscular noncontractile tissue amount evaluated by the echo intensity of B-mode ultrasonography. The critical sample size was estimated to be 59. Hence, 60 participants of both sexes, including 30 older (64–83 years) individuals, were recruited to the study. Their physical characteristics and physical activities are summarized in Table 1. None of the participants had orthopedic or neurological disorders, muscle soreness, or fatigue at the time of measurement, and they were asked to refrain from eating, drinking, or bathing for 1 h and from strenuous exercise for 24 h preceding the experiments for accuracy of S-BIS, torque, and neural activity measurements. All participants were given instructions about the purpose and risks of the study and were required to give written informed consent. The experimental procedure was approved by the ethics committee of the Shibaura Institute of Technology. This study was performed in accordance with the Declaration of Helsinki.

**Table 1:** Physical characteristics, habitual daily activities, variables of segmental bioelectrical impedance spectroscopy, and neuromuscular properties

	Young (*n* = 29)					Older (*n* = 30)					Main effect of age	Effect size (*η*^2^)	
	Mean	SD	Min	Max	95% *CI*	Mean	SD	Min	Max	95% *CI*	*P*	Age	Sex
Physical characteristics													
Age (years)	23	3	21	33	22–24	72	5	64	83	70–74			
Height (cm)	165.3	8.4	152.6	181.2	162.1–168.5	159.8	9.0	141.3	176.4	156.3–163.2	**< 0.001**	0.086	0.571
Weight (kg)	59.8	11.9	40.3	86.3	55.3–64.3	60.1	11.9	38.2	87.2	55.1–64.1	0.808	0.001	0.449
BMI (kg/m^2^)	21.7	2.8	16.3	29.1	20.6–22.8	23.4	3.6	17.9	32.7	21.9–24.6	**0.034**	0.069	0.112
Leg length (cm)	37.3	2.7	32.5	41.5	36.3–38.3	36.0	2.5	32.5	40.5	35.0–36.9	**0.018**	0.056	0.414
Habitual daily activities													
Light (min/day)	621	115	465	857	577–665	715	121	460	947	670–760	**0.003**	0.137	0.066
Moderate (min/day)	67	23	31	145	59–76	56	19	17	99	49–63	**0.036**	0.071	0.076
Vigorous (min/day)	3	4	0	17	1–4	1	2	0	8	0–1	**0.003**	0.146	0.007
S-BIS variables													
ICW (mL)	894.7	265.4	411.7	1323.6	793.7–995.6	773.5	193.9	461.1	1262.9	701.0–845.9	**0.007**	0.060	0.509
ICW/TW	0.710	0.033	0.633	0.762	0.697–0.723	0.661	0.039	0.567	0.736	0.647–0.676	**< 0.001**	0.309	0.151
Explosive muscle strength													
RTD_0–50_ (Nm/s)	145.0	74.1	30.3	288.8	116.8–173.2	87.7	51.6	27.0	198.0	68.4–107.0	**0.001**	0.170	0.055
RTD_50–100_ (Nm/s)	431.7	225.9	113.1	882.4	345.8–517.6	271.3	156.6	65.2	584.5	212.9–329.8	**0.001**	0.146	0.139
RTD_100–200_ (Nm/s)	382.4	143.9	84.0	681.0	327.7–437.2	287.0	125.7	100.4	511.7	240.1–334.0	**0.003**	0.109	0.233
nRTD_0–50_ (%/s)	115.6	50.3	18.3	201.5	96.4–134.7	81.1	40.1	22.1	169.0	66.1–96.0	**0.006**	0.129	0.000
nRTD_50–100_ (%/s)	334.9	135.9	105.3	554.4	283.3–386.8	247.3	114.3	53.2	498.8	204.6–290.0	**0.009**	0.110	0.035
nRTD_100–200_ (%/s)	297.5	70.9	110.0	430.2	270.5–324.5	263.6	88.2	106.8	408.3	230.6–296.4	0.109	0.043	0.054
Maximal muscle strength													
PT_MVC_ (Nm)	111.8	37.7	38.2	184.8	97.4–126.1	88.1	25.0	40.0	140.7	78.8–97.4	**0.001**	0.118	0.311
Twitch contractile properties													
PT_twitch_ (Nm)	21.4	5.6	10.2	34.4	19.3–23.5	17.1	4.1	11.0	27.0	15.6–18.7	**< 0.001**	0.159	0.190
RTD_twitch_ (Nm/s)	176.6	50.1	71.5	298.9	157.5–195.7	125.4	31.1	77.4	200.0	113.8–137.0	**< 0.001**	0.274	0.187
TPT_twitch_ (s)	0.122	0.012	0.098	0.164	0.118–0.127	0.136	0.010	0.110	0.159	0.133–0.140	**< 0.001**	0.293	0.045
Neuromuscular activity													
VA (%)	87.2	14.4	50.0	100.0	81.7–92.6	82.9	16.1	30.9	99.5	76.9–88.9	0.297	0.018	0.060
nRMS-MVC (%M_max_)	3.59	2.01	1.30	8.95	2.83–4.35	3.32	1.78	0.98	6.89	2.66–3.98	0.579	0.004	0.277
nRMS-RTD_0-50_ (%M_max_)	1.49	0.98	0.27	3.32	1.12–1.87	1.44	1.00	0.14	4.76	1.05–1.81	0.835	0.001	0.029
nRMS-RTD_50-100_ (%M_max_)	2.90	1.57	0.75	6.54	2.29–3.49	2.81	1.66	0.33	6.43	2.18–3.42	0.859	0.001	0.115
nRMS-RTD_100-200_ (%M_max_)	3.05	1.62	0.79	7.68	2.44–3.67	3.03	1.97	0.67	8.07	2.29–3.77	0.995	0.000	0.130

*BMI* body mass index, *CI* confidence interval, *ICW* intracellular water, *Max* maximum, *Min* minimum, *MVC* maximal voluntary contraction, *nRMS* normalized root mean square, *nRTD* normalized rate of torque development, *PT* peak torque, *RTD* rate of torque development, *SD* standard deviation, *TPT* time-to-peak torque, *VA* voluntary activation, *BMI* body mass index, *CI* confidence interval, *ICW* intracellular water, *Max* maximum, *Min* minimum, *MVC* maximal voluntary contraction, *nRMS* normalized root mean square, *nRTD* normalized rate of torque development, *PT* peak torque, *RTD* rate of torque development, *SD* standard deviation, *TPT* time-to-peak torque, *VA* voluntary activation

### Habitual physical activities

The participants were asked to continue their routine daily activities while wearing an activity monitor (Active style Pro HJA-750C; Omron Health Care, Japan) for 10 days except when bathing or sleeping. Analyses were performed on data collected when the participants wore the device for at least 500 min a day. The wearing time was recorded using written self-reports. In accordance with the criteria used in a previous study [15], we ascertained that the number of days for analyses should be at least 4. The mean number of days was 9, ranging from 4 to 10. Physical activity was divided into three levels based on the following metabolic equivalents (METs): light (< 3.0 METs), moderate (3.0–6.0 METs), and vigorous intensities (> 6.0 METs) [16].

### Experimental procedure

**Fig. 1:**
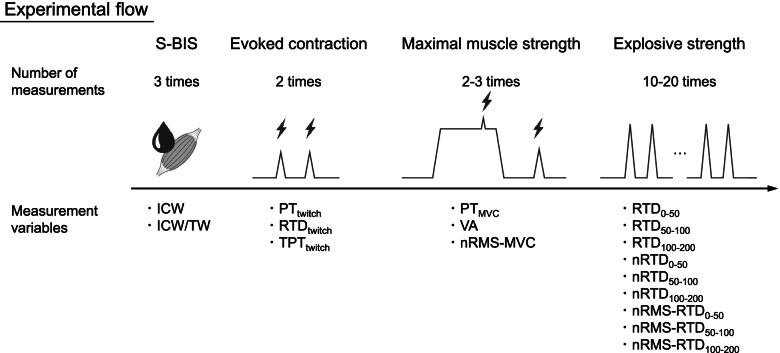
Schematic representation of the experimental flow. Lightning bolt symbols mean electrical stimulations. ICW, intracellular water; ICW/TW, intracellular-to-total water ratio; MVC, maximal voluntary isometric contraction; nRMS, root-mean-square value of electromyographic activity normalized by M-wave amplitude; PT, peak torque; RTD, rate of torque development; S-BIS, segmental bioelectrical impedance spectroscopy; TPT, time-to-peak torque; VA, voluntary activation. The number written in subscript (0–50, 50–100, and 100–200) represents time intervals for RTD analysis. Zero means the onset of contraction

Room temperature was kept at about 23 °C. The experimental flow is shown in Fig. 1. The procedure was briefly described as follows. The S-BIS variables were determined using a bioelectrical impedance analyzer (SFB7; ImpediMed, Australia) prior to all muscle strength measurements to avoid any influence of fluid shift, such as from muscle swelling, due to exercise-induced vasodilation. Then, measurement of evoked contraction of the plantar flexors was conducted by electrical stimulation in order to evaluate the twitch contractile properties. The participants performed maximal voluntary isometric contractions (MVCs) as a maximal strength measurement. Voluntary activation (VA) was also evaluated using the twitch interpolation technique during the MVC task. Lastly, explosive strength of the plantar flexors was measured to calculate RTD. During the all-muscle strength measurements (i.e., electrically evoked muscle strength, maximal muscle strength, and explosive muscle strength), neuromuscular activity of the plantar flexors was also obtained using a surface electromyography (EMG) system. The torque and EMG signals were synchronized and stored on a personal computer using LabChart software (ver.8; ADInstruments, Sydney, Australia) through an A/D converter (PowerLab 16/35; ADInstruments) at 2 kHz sampling frequency and were filtered with 500 Hz lowpass and 20–450 Hz bandpass digital filters, respectively.

### S-BIS variables

Current injection electrodes (20 mm × 20 mm, Red Dot; 3M, USA) were attached over the dorsal surfaces of the right hand and foot. Sensing electrodes were placed on the articular cleft between the lateral femoral and tibial condyles and the lateral malleolus of the right leg. The S-BIS variables for the right leg were measured three times with the SFB7 (ImpediMed, Australia) using a logarithmic distribution of 256 frequencies ranging from 3 to 1000 kHz. Before the measurement, the participants lay supine for 10 min on a stretching mat to avoid the influence of immediate whole-body fluid shift due to posture change from standing. During the measurement, the upper and lower limbs were not in contact with the body or the other limbs, and the participants were fully relaxed. Using the software supplied with the bioelectrical impedance analyzer, the resistances of the ECW (*R*_ECW_) and TW (*R*_TW_) compartments were determined by extrapolation after fitting the spectrum of bioimpedance data to the Cole-Cole model. The resistance of the ICW compartment (*R*_ICW_) was calculated using the following formula: *R*_ICW_ = 1/([1/*R*_TW_]–[1/*R*_ECW_]). The acquired data for each resistance were averaged, and mean values were used for further analyses. Segmental ICW and ECW in the right leg were computed using the following equations: ICW = *ρ*_ICW_ × *L*^2^/*R*_ICW_ and ECW = *ρ*_ECW_ × *L*^2^/*R*_ECW_, where *ρ*_ICW_ (273.9 Ωcm) and *ρ*_ECW_ (47 Ωcm) represent factors for extracellular and intracellular resistivities, respectively, and *L* is the right leg length. TW was calculated as the sum of ICW and ECW, and ICW/TW was computed by dividing ICW by TW.

### EMG electrode placement

In order to evaluate neuromuscular activity of the triceps surae, surface EMG system (Bagnoli 8 EMG System; Delsys Inc., Boston, MA, USA) with pre-amplified bipolar active surface EMG electrodes (electrode shape, parallel bar; size, 1 mm width × 10 mm length; interelectrode distance, 10 mm; DE-2.1, Delsys Inc.) was used. After preparation of the skin (shaving, abrasion, and cleaning with alcohol), the electrodes were placed over the muscle bellies of the medial gastrocnemius, lateral gastrocnemius, and soleus. The electrode locations were at 30% of the leg length (distance between the articular cleft between the lateral femoral and tibial condyles and the lateral malleolus) for the medial and lateral gastrocnemii and midway between the distal myotendinous junctions of the lateral gastrocnemius and the soleus for the soleus. The direction of the electrodes was carefully aligned with the fascicle direction of each muscle using an ultrasonographic device (ACUSON S2000; Siemens Medical Solutions, Ann Arbor, MI, USA). The ground electrode was attached on the lateral malleolus of the left foot.

### Twitch contractile properties and M-wave

Each participant lay supine on the bed of a dynamometer (CON-TREX MJ; Physiomed, Schnaittach, Germany). The hips, knees, and ankles were at the anatomical positions, and the right foot was fixed to the dynamometer foot plate with nonelastic straps. The participants were asked to maintain this posture throughout the all-muscle strength measurements, i.e., electrically evoked muscle strength, maximal muscle strength, and explosive muscle strength. The rotational axes of the dynamometer and ankle joint were visually aligned. In order to percutaneously stimulate the tibial nerve, a cathode (20 mm × 20 mm, Red Dot^;^ 3M) and anode (40 mm × 50 mm, Natus® Disposable Adhesive Electrodes; Natus Manufacturing Limited, Ireland) were attached on the popliteal fossa and the ventral aspect of the thigh, respectively. Rectangular pulses of 200 μs were delivered using a constant-current variable voltage stimulator (DS7AH; Digitimer Ltd., UK). Stimulus intensity was set at 1.2 times the minimum electrical current at which twitch torque reached a plateau.

**Fig. 2:**
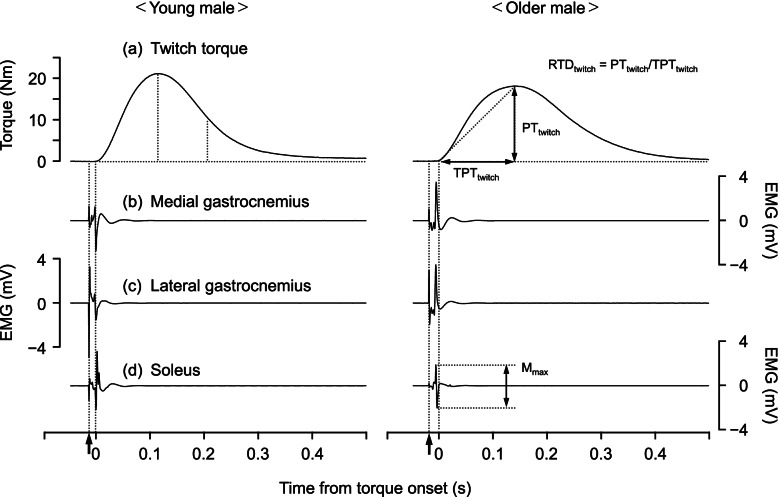
Representative data of torque and electromyography (EMG) signals during twitch contraction in young and older male participants and schematic explanation of measurement variables. **a** Plantar flexion torque, **b** EMG activity of the medial gastrocnemius, **c** EMG activity of the lateral gastrocnemius, and **d** EMG activity of the soleus. Black arrows represent the onset of electrical stimulation. M_max_, peak-to-peak amplitude of M-wave; PT, peak torque; RTD, rate of torque development; TPT, time-to-peak torque

From the twitch response, peak torque (PT_twitch_), RTD (RTD_twitch_), and time-to-peak torque (TPT_twitch_) were determined (Fig. 2). PT_twitch_ was calculated as the difference between the maximal value of the evoked plantar flexion torque and torque at the onset of the evoked contraction (i.e., resting torque). Onset was determined manually as the time point at which the first increase of evoked torque was observed. TPT_twitch_ was defined as the interval between the onset and occurrence PT_twitch_. RTD_twitch_ was computed by dividing PT_twitch_ by TPT_twitch_. The peak-to-peak amplitude of the M-wave (M_max_) elicited by electrical stimulation was calculated for each muscle of the triceps surae. To eliminate the influence of digital filtering on M-wave deformation and to accurately evaluate M_max_, the EMG signal without the bandpass digital filter was used for analysis of the M-wave. For these variables, mean values calculated from the two twitch responses were used for further analyses.

### Maximal muscle strength and VA

After several submaximal contractions as a warm-up, the participants performed two MVCs for 4 s with a 1-min rest period between contractions. When the difference in measured peak torque between the two trials was more than 10% of the highest value, a third trial was conducted. For VA measurement, single electrical stimulations were imposed about 3 s after the start and end of the MVC. The stimulation intensity and duration for VA were identical to those for the twitch contractions.

Peak torque during MVC measurement (PT_MVC_) was calculated as the difference between the maximal value of the voluntary plantar flexion torque and the mean value of resting torque for 1 s. The highest value of PT_MVC_ among the two or three trials was used for further analyses. VA was calculated as (1−[superimposed twitch torque/potentiated resting twitch torque]) × 100 (%). Root-mean-square (RMS) values of the EMG activity of the triceps surae during MVC were determined over a 500-ms period that included the time point of PT_MVC_. VA and the RMS values obtained from the trial containing the highest observed value of PT_MVC_ were used for further analyses. Since neural activation is attenuated by aging [17], the plantar flexion torque produced by full neuromuscular activation (PT_100%_) was calculated as PT_MVC_/VA × 100 [18]. PT_100%_ was used for normalization of RTD (see below). RMS values of EMG activity were normalized by M_max_ for each muscle to give normalized RMS (nRMS) values, which were averaged across the three muscles of the triceps surae.

### Explosive muscle strength

Explosive muscle strength measurement was conducted separately from the maximal muscle strength measurement. According to the recommendation of a previous study [3], the participants exerted brief rapid contractions (~1 s) 10 times, with 20-s rest periods between contractions. The participants were asked to generate force as fast and hard as possible without any counter-movement or pre-activation. Trials with counter-movement (> 0.3 Nm torque variation) or pre-activation (> 3% of RMS-EMG during MVC) in the 200 ms prior to onset of contraction and those that did not reach 70% of PT_MVC_ were excluded from analyses. If an individual failed to perform at least three successful trials, they were asked to perform another 10 trials with sufficient rest periods.

**Fig. 3:**
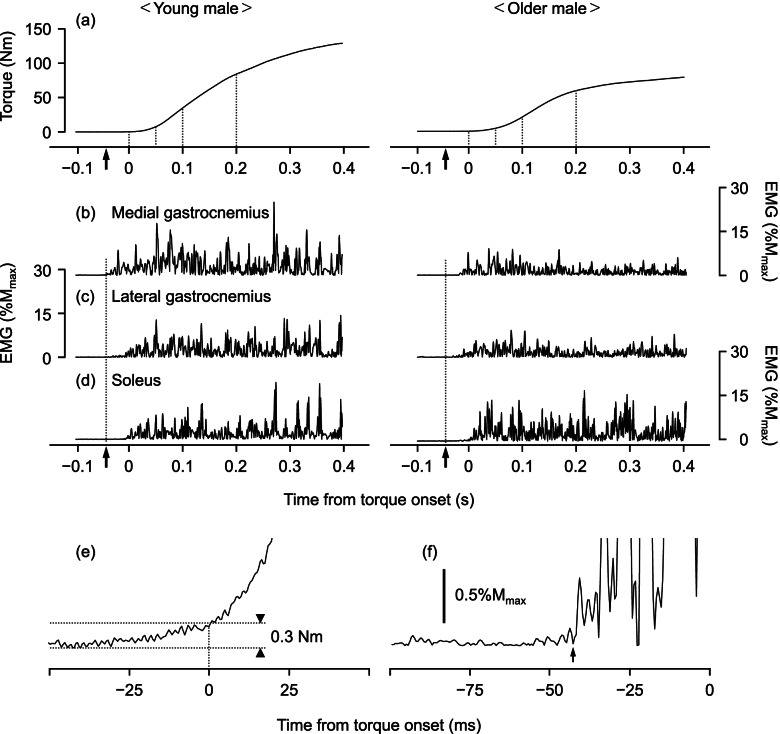
Representative data of torque and electromyography (EMG) signals during explosive muscle strength measurement in young and older male participants and schematic explanation of the onset of torque and EMG activity. **a** Plantar flexion torque, **b** EMG activity of the medial gastrocnemius, **c** EMG activity of the lateral gastrocnemius, **d** EMG activity of the soleus, **e** enlarged view of the time-torque curve around the torque onset in the young participants, and **f** enlarged view of time-EMG activity of the medial gastrocnemius curve around the EMG onset in the young participants. Black arrows represent the onset of EMG activity. M_max_, peak-to-peak amplitude of M-wave

RTD was analyzed for the three trials containing the highest maximal instantaneous RTDs, which were computed from differential waveforms of the time-torque curves in the range from the onset of plantar flexion to 200 ms after onset. For calculation of the instantaneous RTD, a 500 Hz lowpass digital filter was applied to the differential waveform. Onset was determined as the last trough before force deflection above the range of the baseline noise (0.3 Nm) of the time-torque curve [19, 20] (Fig. 3). RTD was calculated as the slope of the time-torque curve over the time intervals of 0–50, 50–100, and 100–200 ms from the onset of contraction. RMS values of EMG activity during the explosive muscle strength measurement were analyzed over the same intervals of RTD from the onset of EMG activity. As with a previous study [19], EMG onset was identified manually as the last trough within the baseline noise envelope from rectified EMG signals. In order to compare rapid force-generating capacity irrespective of differences in potential maximal force generation capacity among the participants, normalized RTDs to PT_100%_ (nRTDs) were calculated for each interval. RMS values of EMG activity were normalized by M_max_ and averaged across the triceps surae for each interval.

### Statistical analyses

The data set of one young female participant was excluded from the entire analyses because the analyzing criteria for RTD were violated. Hence, we analyzed and report the data of 59 participants (30 older and 29 young individuals). In order to compare differences in the measurement values between older and young participants while controlling for sex-related factors, analyses of covariance (ANCOVAs) with a dummy variable for sex as a covariate (women = 0, men = 1) were performed. We report *η*^2^ as the effect size. Similarly, to adjust for sex-related factors, partial correlation analyses were conducted to investigate the associations of S-BIS variables (ICW and ICW/TW) with parameters of explosive muscle strength, maximal strength, twitch contractile properties, and neuromuscular activity for the pooled data (i.e., young and older participants). Additionally, to interpret the relation between ICW/TW and RTD, the previously reported associations of RTD with its determinant factors (maximal strength, twitch contractile properties, and neuromuscular activity in the corresponding time intervals) [12] were also tested using partial correlation analyses with a dummy variable for sex as a control. The partial correlation coefficient (*r*_p_) was regarded as the effect size. We considered effect sizes *η*^2^ ≥ 0.14 as large effects, ≥ 0.06 as medium effects, and ≥ 0.01 as small effects and effect sizes *r*_p_ ≥ 0.5 as large effects, ≥ 0.3 as medium effects, and ≥ 0.1 as small effects [21]. The significance level was set at *α* = 0.05. Descriptive data are presented as means ± SDs.

## Results

### Routine daily activities

Routine daily activities are shown in Table 1. Daily light-intensity physical activity in older participants was higher than that in young participants (*P* = 0.003, *η*^2^ = 0.137). In contrast, moderate- and vigorous-intensity daily activities were lower in older participants than in younger counterparts (*P* ≤ 0.036, *η*^2^ ≥ 0.071).

### Age-related differences in measurement variables

S-BIS variables and neuromuscular properties are shown in Table 1. For S-BIS variables, ICW and ICW/TW were significantly higher in young participants than older participants with medium to large effect sizes (*P* ≤ 0.007, *η*^2^ ≥ 0.060).

For explosive muscle strength, absolute RTDs in all time intervals were significantly larger in young participants than in older counterparts with medium to large effect sizes (*P* ≤ 0.003, *η*^2^ ≥ 0.109). The nRTD_0–50_ and nRTD_50–100_ were also larger in young participants compared with older participants with medium effect sizes (*P* ≤ 0.009, *η*^2^ ≥ 0.110), although nRTD_100–200_ was not significantly different between the age groups (*P* = 0.109, *η*^2^ = 0.043).

Regarding maximal muscle strength, PT_MVC_ was significantly larger in young participants than in older counterparts with a medium effect size (*P* = 0.001, *η*^2^ = 0.118). For twitch contractile properties, PT_twitch_ and RTD_twitch_ were significantly larger in young individuals than older individuals with large effect sizes (*P* < 0.001, *η*^2^ ≥ 0.159). TPT_twitch_ in young participants was significantly shorter than for older participants with a large effect size (*P* < 0.001, *η*^2^ = 0.293).

VA did not differ between young and older participants (*P* = 0.297, *η*^2^ = 0.018). Similarly, no significant differences in nRMS during maximal or explosive muscle strength measurements were observed between the age groups (*P* ≥ 0.579, *η*^2^ ≤ 0.004).

### Partial correlations of S-BIS variables with explosive muscle strength, maximal strength, twitch contractile properties, and neuromuscular activity

Table 2 shows the partial correlations (controlled for sex-related factors) of S-BIS variables with neuromuscular properties. For explosive muscle strength, significant positive correlations of ICW and ICW/TW with absolute RTDs were observed in all time intervals with medium effect sizes (*r*_p_ ≥ 0.336, *P* ≤ 0.010). ICW/TW also correlated positively with nRTDs in all time intervals with medium effect sizes (*r*_p_ ≥ 0.304, *P* ≤ 0.020), but ICW did not (*r*_p_ ≤ 0.247, *P* ≥ 0.062).

**Table 2:** Partial correlation coefficients (*r*_p_) of variables of segmental bioelectrical﻿ impedance spectroscopy with neuromuscular properties (*n* = 59)

	S-BIS variables			
	ICW		ICW/TW	
	*r*_p_	*P*	*r*_p_	*P*
Explosive muscle strength				
RTD_0–50_	**0.336**	0.010	**0.377**	0.004
RTD_50–100_	**0.364**	0.005	**0.397**	0.002
RTD_100–200_	**0.419**	0.001	**0.408**	0.001
nRTD_0–50_	0.169	0.204	**0.311**	0.017
nRTD_50–100_	0.225	0.090	**0.354**	0.006
nRTD_100–200_	0.247	0.062	**0.304**	0.020
Maximal muscle strength				
PT_MVC_	**0.460**	< 0.001	**0.378**	0.003
Twitch contractile properties				
PT_twitch_	**0.646**	< 0.001	**0.504**	< 0.001
RTD_twitch_	**0.654**	< 0.001	**0.567**	< 0.001
TPT_twitch_	**−0.276**	0.036	**−0.392**	0.002
Neuromuscular activities				
VA	0.196	0.141	0.111	0.407
nRMS-MVC	−0.033	0.803	0.031	0.818
nRMS-RTD_0–50_	−0.015	0.914	−0.005	0.973
nRMS-RTD_50–100_	−0.027	0.840	0.029	0.830
nRMS-RTD_100–200_	−0.045	0.735	0.023	0.862

*ICW* intracellular water, *MVC* maximal voluntary contraction, *nRMS* normalized root mean square, *nRTD* normalized rate of torque development, *PT* peak torque, *RTD* rate of torque development, *TPT* time-to-peak torque, *VA* voluntary activation

Significant positive correlations of ICW and ICW/TW with PT_MVC_ were observed with medium effect sizes (*r*_p_ ≥ 0.378, *P* ≤ 0.003). Regarding twitch contractile properties, there were significant positive correlations of ICW and ICW/TW with both PT_twitch_ and RTD_twitch_ with large effect sizes (*r*_p_ ≥ 0.504, *P* < 0.001). ICW and ICW/TW were significantly negatively correlated with TPT_twitch_ with small to medium effect sizes (*r*_p_ ≤ −0.276, *P* ≤ 0.036).

For neuromuscular activity, no S-BIS variable correlated with VA (*r*_p_ ≤ 0.196, *P* ≥ 0.141). Also, no significant correlation was seen between S-BIS variables and nRMSs during maximal- and explosive-muscle strength measurements (|*r*_p_| ≤ 0.045, *P* ≥ 0.735).

### Partial correlations of explosive muscle strength with maximal muscle strength, twitch contractile properties, and neuromuscular activity

Partial correlations (controlled for sex-related factors) of explosive muscle strength with maximal strength, twitch contractile properties, and neuromuscular activity are shown in Table 3. Briefly, as reported previously,^12^ RTDs positively correlated with maximal muscle strength and nRMSs in the corresponding time intervals with large effect sizes (*r*_p_ ≥ 0.527, *P* < 0.001). Significant correlations between RTDs and twitch contractile properties were also found for most of the combinations except between RTD_100–200_ and TPT_twitch_, between nRTD_0–50_ and PT_twitch_, or between nRTD_100–200_ and twitch contractile properties (PT_twitch_, RTD_twitch_, and TPT_twitch_).

**Table 3:** Partial correlation coefficients of explosive muscle strength with influencing factors (*n* = 59)

	Explosive muscle strength											
	RTD_0–50_		RTD_50–100_		RTD_100–200_		nRTD_0–50_		nRTD_50–100_		nRTD_100–200_	
	*r*_p_	*P*	*r*_p_	*P*	*r*_p_	*P*	*r*_p_	*P*	*r*_p_	*P*	*r*_p_	*P*
Maximal muscle strength												
PT_MVC_	**0.539**	< 0.001	**0.644**	< 0.001	**0.860**	< 0.001	−	−	−	−	−	−
Twitch contractile properties												
PT_twitch_	**0.480**	< 0.001	**0.522**	< 0.001	**0.525**	< 0.001	0.252	0.056	**0.307**	0.019	0.199	0.135
RTD_twitch_	**0.531**	< 0.001	**0.558**	< 0.001	**0.481**	< 0.001	**0.311**	0.017	**0.352**	0.007	0.177	0.185
TPT_twitch_	**−0.361**	0.005	**−0.313**	0.017	−0.080	0.551	**−0.312**	0.017	**−0.272**	0.039	−0.021	0.875
Neuromuscular activities												
nRMS-RTD_0–50_	**0.607**	< 0.001	−	−	−	−	**0.648**	< 0.001	−	−	−	−
nRMS-RTD_50–100_	−	−	**0.569**	< 0.001	−	−	−	−	**0.624**	< 0.001	−	−
nRMS-RTD_100–200_	−	−	−	−	**0.527**	< 0.001	−	−	−	−	**0.557**	< 0.001

*MVC* maximal voluntary contraction, *nRMS* normalized root mean square, *nRTD* normalized rate of torque development, *PT* peak torque, *RTD* rate of torque development, *TPT* time-to-peak torque, *VA* voluntary activation

## Discussion

The present study aimed to cross-sectionally examine the association of age-related differences in ICW/TW with RTD in young and older individuals. With the exception of nRTD_100–200_, significant differences in ICW/TW and all absolute and normalized RTDs values between the age groups were observed. Positive associations of ICW/TW with absolute and normalized RTDs values in all time intervals were also evident. These results support our hypothesis and suggest that age-related decreases in the ICW/TW reflect decreasing RTD.

We observed lower ICW and ICW/TW in older participants compared with young participants (Table 1). Yamada et al. [8] reported similar age-related decreases in ICW and ICW/TW. ICW and ICW/TW represent muscle cell mass and occupancy of muscle cells within whole muscle, respectively [10]. Decreases in the number and size of muscle fibers and the spread of extracellular space are typical aging events of skeletal muscle [6]. Hence, age-associated differences in ICW and ICW/TW observed in the older participants of this study were indicative of typical anatomical characteristics of older skeletal muscles.

With the exception for nRTD_100–200_, absolute and normalized RTD values of voluntary plantar flexion in older participants were consistently lower than in younger counterparts (Table 1). A previous study [12] claimed that maximal muscle strength, twitch contractile properties, and neuromuscular activity determine RTD. Similarly, we found significant correlations of RTD with maximal muscle strength, twitch contractile properties, and neuromuscular activity in most of the combinations (Table 3). Furthermore, differences in PT_MVC_, PT_twitch_, RTD_twitch_, and TPT_twitch_ between the age groups were evident (Table 1). These results indicate that the age-related difference in RTD observed in this study was due to maximal muscle strength and twitch contractile properties but not neuromuscular activity.

ICW correlated positively with absolute RTDs but not with the normalized RTDs (Table 2). Since ICW can be equated to muscle cell mass [10], ICW is related to maximal muscle strength, as shown in the present and previous studies [8]. In addition, it is known that the absolute RTD value is affected by maximal muscle strength [12], as the present results also show (Table 3). Hence, it is reasonable that ICW associated with absolute RTD but not with normalized RTD.

Significant positive correlations of ICW/TW with absolute and normalized RTD values were found. To the best of our knowledge, this is the first study to elucidate these relations. These associations may be due to a potential connection between the age-related increase in extracellular space [8] and decrease in type II fiber content [5], which both occur in typical skeletal muscle aging. Muscle fiber with a higher content of myosin heavy chain II shows superior RTD and shorter TPT [4]. In the present study, lower ICW/TW and RTD values and longer TPT_twitch_ in older volunteers were evident (Table 1). Also, there were associations among ICW/TW, RTD, and TPT_twitch_ (Tables 2 and 3). These results suggest that the decrement in ICW/TW with age might reflect age-related changes in intrinsic contractile properties, resulting in the observed association between age-related differences in ICW/TW and RTD. However, while a significant correlation between ICW/TW and nRTD_100–200_ was observed (Table 2), there was no difference in nRTD_100–200_ between the age groups, and it did not relate to twitch contractile properties (Tables 1 and 3). These results imply that the association of ICW/TW with RTD is attributable to several factors other than intrinsic contractile properties. Although these factors were not assessed in this study, impairment of lateral force transmission, which is suggested to occur by increased thickness of the extracellular matrix [22], may be one of the reasons. Future studies are warranted to explore other potential mechanisms in the association of ICW/TW with RTD.

Our results demonstrate the potential usefulness of ICW/TW as an index of the age-associated decrease in rapid force-generating capacity. ICW/TW can be measured quickly (few seconds or less), noninvasively, and painlessly using a portable and inexpensive S-BIS device. This also means that ICW/TW can predict rapid force-generating capacity without the need for direct muscle strength measurement using a dynamometer. Especially for older adults, muscle contractions with maximal effort may cause unexpected muscle injuries. Furthermore, although individuals with dementia have an increased risk of developing sarcopenia [23], strength measurements in these patients can be difficult to conduct, making sarcopenia diagnosis difficult. Since RTD has been suggested to relate to the functional performance of many daily tasks [24] and steeply decreases with age [1], the quick and safe measurement of ICW/TW to evaluate rapid force-generating capacity without muscle contraction may be a valuable tool in certain situations, such as for screening tests in elderly nursing homes.

The present study has several limitations. First, the S-BIS variables were calculated based on intra- and extracellular water content within the entire leg muscles rather than just the plantar flexors. Therefore, age-associated differences in the quantity and quality of nontarget muscle groups (e.g., the dorsiflexors) may have contributed to the observed association of S-BIS variables with RTD values. However, age-related decreases in muscle strength [25] and size [26] are not believed to vary between the plantar flexors and dorsiflexors. Therefore, any influence of nontarget muscles on the interpretation of these results should be small. Second, contrary to a previous report [15], the indices of neuromuscular activity (VA, nRMS-MVC, and nRMS-RTD) did not vary between young and older participants. This may have been because the older participants in this study were relatively active. For instance, mean values of moderate- to vigorous-intensity physical activities (> 3 METs) in the Japanese older population were reported to be 20.4 ± 19.2 (*n* = 401 [women = 178], age = 71.1 ± 4.3 years [27];) and 17.4 min/day (*n* = 220 [women = 129], age = 65–84 years [28];), respectively, whereas the older participants here achieved 57 ± 19 min/day. RTD was clearly associated with EMG activity during the assessed time intervals (*r*_p_ = 0.527–648, *P* < 0.001; Table 3), but no relation between ICW/TW and nRMS-RTD values was observed for the same time intervals (Table 2). While these limitations may weaken the observed association of ICW/TW with RTD, they were only evident with medium effect sizes and may therefore actually support the present findings. Collectively, age-related differences in ICW/TW may become useful indicators of RTD.

## Conclusions

Age-related differences were observed in S-BIS variables, explosive- and maximal-muscle strength, and twitch contractile properties, but not in EMG activity. ICW/TW was positively related to absolute and normalized RTDs. Additionally, ICW/TW was associated with twitch contractile properties, but not with EMG activity during rapid force production. These results indicate that ICW/TW may be a useful predictor of the age-related decrease in RTD, and that the decrease in ICW/TW with age may reflect age-associated changes in intrinsic contractile properties.

## Data Availability

The datasets used and/or analyzed during the current study are available from the corresponding author on reasonable request.

## Abbreviations

ANCOVA: Analysis of covariance
ECW: Extracellular water
EMG: Electromyography
ICW: Intracellular water
ICW/TW: Ratio of intracellular water to total water
METs: Metabolic equivalents
M_max_: Peak-to-peak amplitude of M-wave
MVC: Maximal voluntary isometric contraction
nRMS: Normalized root mean square
nRTD: Normalized rate of torque development
PT: Peak torque
RMS: Root mean square
RTD: Rate of torque development
S-BIS: Segmental bioelectrical impedance spectroscopy
TPT: Time-to-peak torque
TW: Total water
VA: Voluntary activation
